# Calcium dysregulation via L-type voltage-dependent calcium channels and ryanodine receptors underlies memory deficits and synaptic dysfunction during chronic neuroinflammation

**DOI:** 10.1186/s12974-015-0262-3

**Published:** 2015-03-25

**Authors:** Sarah C Hopp, Heather M D’Angelo, Sarah E Royer, Roxanne M Kaercher, Alexis M Crockett, Linda Adzovic, Gary L Wenk

**Affiliations:** Departments of Neuroscience, Ohio State University, Columbus, OH 43210 USA; Department of Psychology, Ohio State University, 1835 Neil Ave, Columbus, OH 43210 USA

**Keywords:** Calcium, Neuroinflammation, Ryanodine receptors, L-type voltage-dependent calcium channels, Spatial memory

## Abstract

**Background:**

Chronic neuroinflammation and calcium (Ca^+2^) dysregulation are both components of Alzheimer’s disease. Prolonged neuroinflammation produces elevation of pro-inflammatory cytokines and reactive oxygen species which can alter neuronal Ca^+2^ homeostasis via L-type voltage-dependent Ca^+2^ channels (L-VDCCs) and ryanodine receptors (RyRs). Chronic neuroinflammation also leads to deficits in spatial memory, which may be related to Ca^+2^ dysregulation.

**Methods:**

The studies herein use an *in vivo* model of chronic neuroinflammation: rats were infused intraventricularly with a continuous small dose of lipopolysaccharide (LPS) or artificial cerebrospinal fluid (aCSF) for 28 days. The rats were treated with the L-VDCC antagonist nimodipine or the RyR antagonist dantrolene.

**Results:**

LPS-infused rats had significant memory deficits in the Morris water maze, and this deficit was ameliorated by treatment with nimodipine. Synaptosomes from LPS-infused rats had increased Ca^+2^ uptake, which was reduced by a blockade of L-VDCCs either *in vivo* or *ex vivo*.

**Conclusions:**

Taken together, these data indicate that Ca^+2^ dysregulation during chronic neuroinflammation is partially dependent on increases in L-VDCC function. However, blockade of the RyRs also slightly improved spatial memory of the LPS-infused rats, demonstrating that other Ca^+2^ channels are dysregulated during chronic neuroinflammation. Ca^+2^-dependent immediate early gene expression was reduced in LPS-infused rats treated with dantrolene or nimodipine, indicating normalized synaptic function that may underlie improvements in spatial memory. Pro-inflammatory markers are also reduced in LPS-infused rats treated with either drug. Overall, these data suggest that Ca^+2^ dysregulation via L-VDCCs and RyRs play a crucial role in memory deficits resulting from chronic neuroinflammation.

## Introduction

Chronic neuroinflammation is a component of normal aging and may contribute to age-related cognitive decline as well as neurodegenerative disorders such as Alzheimer’s disease (AD; [1]). One of the primary effector cells of neuroinflammation are microglia, the resident macrophages of the central nervous system. Normally, microglia contribute to normal neuronal function, but chronic microglia activation can cause damage to nearby neurons [2]. Several aspects of AD can be replicated by chronic infusion of lipopolysaccharide (LPS) into the fourth ventricle of young rats ([3,4]. Chronic neuroinflammation in young rats impairs performance in a variety of memory tasks [5] and such memory impairments are associated with long-term potentiation (LTP) deficits [6].

Ca^+2^ handling is altered in non-neuronal tissues derived from AD patients and family members [7]. Epidemiological studies have shown that the use of L-type voltage-dependent calcium channel (L-VDCC) antagonists by patients with cardiovascular conditions is associated with a reduced incidence of AD in [8] and patients treated with the L-VDCC antagonist nimodipine have improved cognitive scores compared to placebo-treated patients [9]. Ryanodine receptors (RyRs) may represent a novel target for treatment of Alzheimer’s disease. RyR expression is altered in patients with AD and mild cognitive impairment [10], and patients with sporadic AD both have L-VDCC and RyR mutations that interact to increase and have amyloid deposition [11], demonstrating the importance of these two channels in the AD pathology. In addition to normalizing calcium dysregulation, targeting of RyRs and L-VDCCs *in vitro* is anti-inflammatory [12-14], and previous epidemiological studies have revealed that use of other anti-inflammatory drugs such as nonsteroid anti-inflammatory drugs (NSAIDs) also reduces Alzheimer’s disease incidence [15].

Neuroinflammation and neuronal Ca^+2^ dysregulation may interact, synergistically leading to memory deficits. Neuroinflammation increases glutamatergic activity by suppression of glutamate transport ([13,16-18]) while potentiating activity of N-methyl D-aspartate receptors (NMDARs; [19-21]). Similarly, pro-inflammatory cytokines and nitric oxide (NO) can increase the function of L-VDCCs [22] and RyRs [23,24]. Both NMDAR-dependent and L-VDCC-dependent LTP are disrupted during chronic neuroinflammation [25]. Additionally, the function of RyRs and L-VDCCs are linked not only to each other but also to the function of NMDARs [26-28]. RyRs interact with NMDARs by amplifying NMDAR Ca^+2^ signals [26], while L-VDCCs can decrement relevant NMDAR event-related signaling by lengthening the after hyperpolarization [28]. Overall, these data suggest that these channels can all act synergistically to increase intracellular Ca^+2^ concentration during neuroinflammation and disrupt normal processes that underlie memory. Increased intracellular Ca^+2^ could lead to memory deficits via dysregulated activation of Ca^+2^-dependent kinases and subsequent production of immediate early genes (IEGs) such as activity-regulated cytoskeleton-associated protein (*Arc)*.

Overall, these data have led to the following hypotheses. 1) If neuroinflammation leads to increases in intracellular Ca^+2^ levels, then increased *Arc* production should be observed in tissue from rats chronically infused with LPS, since Arc induction is Ca^+2^ dependent [29]. Furthermore, transport of Ca^+2^ should be observed directly in synaptosomes generated from the hippocampus of these rats. 2) If neuroinflammation-induced memory deficits are due to increased intracellular Ca^+2^ and dysregulation of L-VDCCs and/or RyRs, then pharmacological blockade of these channels should improve spatial memory deficits and normalize Ca^+2^ levels and activity of Ca^+2^-dependent markers.

## Methods

### Subjects and surgical procedures

The subjects were male F-344 (Harlan, Indianapolis, IN, USA) rats, 3 months old, individually housed with *ad libitum* access to food and water and maintained on a reverse 12/12 light/dark cycle with lights off at 8 AM. Artificial cerebrospinal fluid (aCSF, 140 mM NaCl, 3.0 mM KCl, 2.5 mM CaCl2, 1.0 mM MgCl2, 1.2 mM Na2HPO4, pH 7.4; *n* = 40) or LPS (Sigma, St. Louis, MO, USA, *Escherichia coli* serotype 055:B5 TCA extraction, 1.0 mg/ml dissolved in aCSF, *n* = 43) was loaded into an osmotic minipump (Alzet model #2004, with a rate of 0.25 μl/hr, Durect Corp., Cupertino, CA, USA) and infused into the brain for 28 days via a cannula surgically implanted into the fourth ventricle as previously described [30]. The day after the osmotic minipump was implanted, rats began to receive daily subcutaneous drug injections at a volume of 1 ml/kg per day with a vehicle (polyethylene glycol 300, Thermo Fisher Scientific, Waltham, MA, USA), dantrolene sodium salt (5 mg/kg/day, Sigma), or nimodipine (5 mg/kg/day, Sigma), resulting in six group + drug treatment groups (aCSF + vehicle, *n* = 14; aCSF + dantrolene, *n* = 13; aCSF + nimodipine, *n* = 13; LPS + vehicle, *n* = 16; LPS + dantrolene, *n* = 14; LPS + nimodipine, *n* = 14). Body weights were monitored daily, and rats were given saline injections and supplemental food postoperatively to prevent dehydration and weight loss. This research was carried out in accordance with the National Institutes of Health Guide for the Care and Use of Laboratory Animals (NIH Publication No. 80–23) and The Ohio State University Institutional Animal Care and Use Committee.

### Behavioral testing

The rats were handled daily for 1 week prior to surgery and daily up until behavioral testing, which took place on the third week following surgery. Spatial learning was assessed in the Morris water maze (MWM), using a 170-cm diameter pool with gray walls surrounded by multiple visual distal and proximal cues. The water was maintained at room temperature (RT; 21°C to 22°C). During the hidden platform portion of the task, a circular escape platform was present in a consistent location and submerged 2.5 cm below the water surface. The rats were tracked using overhead cameras and Noldus Ethovision 3.1 tracking and analysis stem (Noldus, Lessburg, VA, USA). On the first day, rats were placed on the hidden platform for 30 s prior to the trial. Each rat performed six trials per day separated by a 60-min inter-trial interval for four consecutive days. The rat was released into the water at one of six randomized locations which were varied such that rats were could not take the same path to the hidden platform more than once per day. After the rat located the hidden platform or swam for a maximum of 60 s, it was placed on the platform for 30 s. After the last training trial on the fourth day, the rats were tested in a 60-s probe trial where the platform was removed from the pool. Finally, at the end of the fourth day, the rats were tested in a visual platform test where the platform was moved to a new quadrant and raised 2 cm above the surface of the water in order to control for any group- or drug-related differences in visual acuity or swimming ability. All rats across all groups were able to locate the visible platform. Three days after the conclusion of the water maze task, in order to assess expression of the behaviorally induced immediate early gene *Arc*, rats were exposed to a novel context 30 min prior to sacrifice. The novel context was an exploration box (36 × 48 cm) surrounded by proximal and distal visual stimuli.

### Tissue collection

All of the rats were deeply anesthetized using isoflurane prior to sacrifice. One cohort of rats was used for histology (*n* = 6 per each group + drug) and another used for biochemistry (*n* = 7 to 10 per each group + drug). The histology cohort underwent transcardiac perfusion with cold saline containing 1 U/ml heparin followed by 4% paraformaldehyde in 0.1 M phosphate buffer, pH 7.4. The brains were post-fixed overnight in fixative and then stored at 4°C in phosphate-buffered saline (PBS), pH 7.4. The biochemistry cohort was rapidly decapitated and their hippocampi dissected on ice and separated such that the right or left sides were randomly chosen for either protein or gene expression analysis. The hippocampi were stored at −80°C until RNA or protein extraction. A separate cohort of rats was used for ^3^H-radioligand binding assays, cell isolation procedures, and generation of synaptosomes for use in the ^45^Ca^+2^ uptake (*n* = 10/group). For these rats, one hippocampus was used immediately for the generation of synaptosomes.

### Immunohistochemistry

Free-floating coronal sections (40-μm thickness) were generated using a vibratome and stained using standard avidin/biotin-peroxidase-labeling methods as previously described [31]. The rabbit polyclonal antibody against *Arc* (final dilution 1:2,000; Synaptic Systems, Goettingen, Germany) was used to label behaviorally activated neurons; the mouse monoclonal antibody against OX-6 (final dilution 1:200; BD Pharmigen, San Diego, CA, USA) was used to label major histocompatibility complex II (MHC-II) on activated microglia; and the mouse monoclonal antibody against glial fibrillary acid protein (GFAP; final dilution 1:2,000; Millipore Chemicon, Billerica, MA, USA) was used to quantify astrocyte activation. Briefly, endogenous peroxidase activity was quenched and nonspecific binding was blocked with 5% normal goat serum (NGS) in PBS (OX-6 and GFAP) or Tris-buffered saline (TBS) with 5% Tween (*Arc*). Sections were then incubated overnight at 4°C in primary antibody diluted in the same blocking solution. The next day, sections were rinsed and then incubated for 1.5 h at RT in biotinylated secondary antibody from the appropriate species (final dilution 1:200, Vector, Burlingame, CA, USA). Sections were then rinsed and incubated for 1 h at RT with avidin-biotinylated horseradish peroxidase (Vectastain, ABC kit, Vector). After another rinse, sections were incubated with 0.05% 3, 3′-diaminobenzidine tetrahydrochloride (Vector) as a chromogen. The reaction was stopped by rinsing the sections with PBS. No staining was detected in the absence of primary or secondary antibodies. Sections were mounted on superfrost slides, air-dried, dehydrated with a series of ethanol and xylene rinses, and cover slipped with cytoseal (Allan Scientific, Kalamazoo, MI, USA) mounting medium. Images of the hippocampi were captured with light microscopy, stitched together, and analyzed with a Nikon 90i system with a DS-5 M-L1 digital camera using Elements 3.1 Software (Nikon Instruments, Melville, NY, USA). Subfields of interested were determined as previously reported [32]. OX-6 was quantified using automated cell-counting methods as previously described [6]. OX-6 immunoreactive objects larger than 65 mm^2^ were included in analysis, and data are expressed as number of objects per mm^2^. *Arc* and GFAP were quantified using intensity densitometry and data are expressed as % area (of the region of interest).

### RNA isolation and quantitative polymerase chain reaction

Total RNA was isolated from hippocampi with phenol-chloroform extraction using PureZol (Bio-Rad, Hercules, CA) followed by cleanup using NucleoSpin RNA II kits (Machery-Nagel, Düren, Germany) according to the manufacturers’ instructions. RNA quantity was measured using a Synergy Plate reader equipped with a Take-3 plate (Bio-Tek, Winooski, VT, USA) and 1 μg from each sample was used to generate cDNA with iScript reverse transcription Supermix kit (Bio-Rad) using a C1000 Thermal Cycler (Bio-Rad). Selected RNA samples were run without reverse polymerase as a control to ensure no contamination from genomic DNA. Primers were designed using the PrimerQuest software (Integrated DNA Technologies, Coralville, IA, USA; Table 1). Gene expression was quantified using SsoAdvanced Universal SYBR Green (Bio-Rad) quantitative polymerase chain reaction (qPCR) on a CFX96 Real-Time PCR detection system (Bio-Rad). Glyceraldehyde-3-phosphate dehydrogenase (GAPDH) was used as a reference gene. Data were analyzed using the comparative threshold cycle method with results expressed as a fold change versus aCSF + vehicle rats.

**Table 1:** **Primer sequences**

**Target**	**Accession #**	**Forward**	**Reverse**
GAPDH	NM_017008	TGACTCTACCCACGGCAAGTTCAA	ACGACATACTCAGCACCAGCATCA
RyR1	AF112256	TGCGCTCCAACCAGGATCTCATTA	TCACCTCGAAGTACCACTTGCCATAC
RyR2	AF112257	GGACTTGAAGGAACTGACGGAGGAAA	CACTGAGACCAGCATTTGGGTTGTG
RyR3	AF130881	TGGCCTCCTGGCTGTAGTTGTTTA	ACCTGCTCTTACGCCCACATACAT
VDCC a1c	NM_012517	GGCTATGAGTTGCCCTGGGTGTATTT	CGAGCTTTGGCTTTCTCCCTCTCTTT
VDCC a1d	NM_017298	TGCATGACATTGGGCCAGAAATCC	AGTTTCCAAGCAGGGCACCATTTC
IL-1β	NM_031512	ACCTGCTAGTGTGTGATGTTCCCA	AGGTGGAGAGCTTTCAGCTCACAT
TNFα	X66539.1	CTGGCCAATGGCATGGATCTCAAA	AGCCTTGTCCCTTGAAGAGAACCT
TGFβ	NM_021578	TGATACGCCTGAGTGGCTGTCTTT	TTTGCTGTCACAAGAGCAGTGAGC
iNOS	NM_012611	AGTTTCCAAGCAGGGCACCATTTC	TGGGTGTCAGAGTCTTGTGCCTTT
ARC	NM_017134	AGCTACCTGCTGGGAAGGAAGAAA	CTTCTCTGTAAGATAGGCCTCCCACAAC
CD200 ligand	NM_031518	CCTGAACGTGTTTCCCTGGTCTACTT	GTCAAATCCCTCACAGGCTTCCTTCT
CD200 receptor	NM_023953	GCGGCTGAGTCAAGTTGTCCTGATA	TGAAATAGAAGGGCAGCAGAGCAGAG

*GAPDH* glyceraldehyde-3-phosphate dehydrogenase, *RyR* ryanodine receptor, *VDCC* voltage-dependent Ca^+2^ channel, *TNFα* tumor necrosis factor alpha, *TGFβ* transforming growth factor β, *iNOS* inducible nitric oxide synthase, *ARC* activity-regulated cytoskeleton-associated protein.

### ^45^Ca^+2^ synaptosomal uptake

A ^45^Ca^+2^ synaptosomal uptake assay was used in order to determine whether LPS treatment would increase Ca^+2^ uptake and whether such an observed increase could be blocked by L-VDCC antagonism. Rats infused with LPS or aCSF and treated with vehicle or nimodipine were used for this assay. Synaptosomes were generated immediately from freshly dissected hippocampus by Teflon/glass homogenization in 0.32 mM sucrose. Homogenates were centrifuged at 3,000 RPM for 10 min at 4°C, followed by a second centrifugation of the supernatant at 12,000 RPM for 10 min at 4°C. The resulting pellet containing synaptosomes was then resuspended in HEPES buffer (125 mM NaCl, 3.5 mM KCl, 0.4 mM KH_2_PO_4_, 1.2 mM MgSO_4_ 7(H2O), 10 mM d-glucose, 1 mM CaCl_2_, and 20 mM HEPES) and placed on ice until use. An aliquot of each sample was saved for protein quantification using a Bio-Rad protein assay. HEPES buffer with 0.03 mM nimodipine (Nim-HEPES) was prepared for use during the blocked condition. HEPES buffer (60 μl) containing 0.01 μCi/μl of ^45^Ca^+2^ (Perkin Elmer, Boston, MA, USA) was added to an incubation vial followed by 60 μl of either HEPES buffer or Nim-HEPES. Synaptosomes (60 μl) were added last and incubated at RT for 1 min and uptake was stopped by filtration onto 0.45-μm nitrocellulose filters (Millipore, Billerica, MA, USA). Filters were transferred to scintillation vials and filled with scintillation fluid (Formula 989, Perkin Elmer, Boston, MA, USA). Vials were loaded into a liquid scintillation counter (1900 TR Tri Carb, Packard Instrument Company/Perkin-Elmer) and number of decays per minute (DPMs) were counted over 10 min per sample. All samples were run in triplicate, plus blanks and external standards containing 10 μl of the HEPES buffer containing 0.01 μCi/μl of ^45^Ca^+2^. For data analysis, blank values were subtracted from the sample values; DPMs were converted to moles of ^45^Ca^+2^ based on the specific activity of the radioisotope and Curie’s constant. Finally, the moles of ^45^Ca^+2^ were divided by protein content of 60 μl of synaptosomes such that data are presented as moles of ^45^Ca^+2^ per mg protein.

### Statistical analyses

Statistical analyses were conducted using SigmaPlot 12.5 (Systat, San Jose, CA, USA). Analyses of variance (ANOVA) were performed followed by either the Bonferroni correction to counteract the problem of multiple *post hoc* comparisons associated with analysis of the MWM data or Fisher’s protected least significant difference for *post hoc* comparisons for the biochemistry and histology data. Graphs display the mean plus standard error of the mean (SEM). A *P* < 0.05 was considered statistically significant.

## Results

### Blockade of L-VDCCs or RyRs improves spatial memory during chronic neuroinflammation

**Figure 1:**
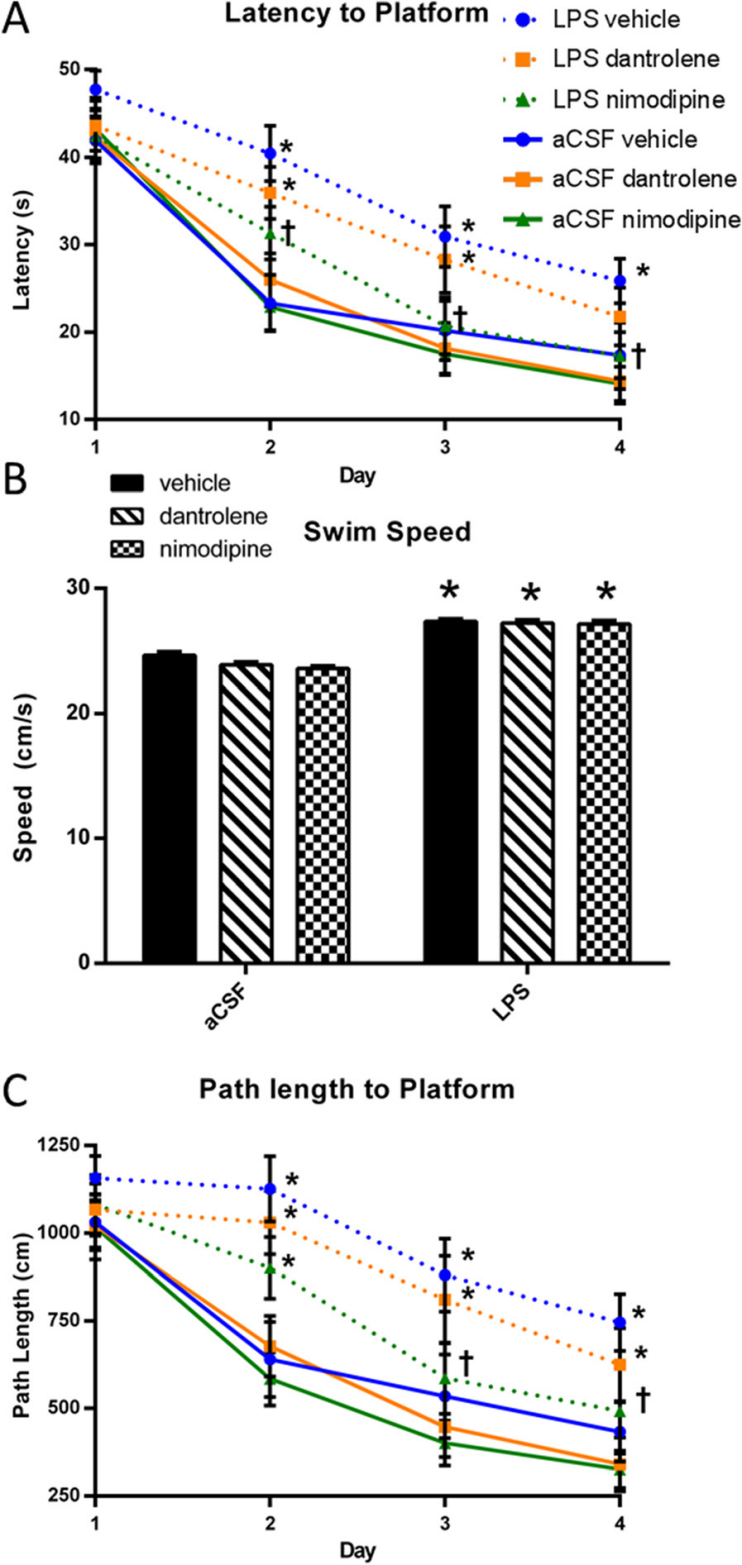
**Morris water maze spatial memory task performance was assessed beginning in the third week of LPS (25 ng/hr) or aCSF infusion and drug treatment with either vehicle, dantrolene (5 mg/kg), or nimodipine (5 mg/kg) with 12 to 15 rats per infusion × drug group.** Performance was assessed by **(A)** path length and **(C)** latency to locate the hidden platform across days of training, as well as average swim speed **(B)** across all trials. Data expressed as mean ± SEM. *Indicates a significant difference from treatment-matched aCSF controls, ^†^indicates a significant difference from LPS + vehicle rats within the LPS infusion group. Significance determined by *P* < 0.05. *LPS* lipopolysaccharide, *aCSF* artificial cerebrospinal fluid.

After 3 weeks of LPS or aCSF infusion and treatment with vehicle, dantrolene, or nimodipine (*n* = 12 to 15/group), we tested the rats’ performance in the MWM (Figure 1). We examined the path length that rats took to locate the hidden platform (Figure 1A), with longer path lengths indicative of a spatial learning deficit. We first performed a three-way ANOVA (infusion group × drug group × day of testing) and then a *post hoc* two-way repeated measures (RM)-ANOVA (combining infusion group and drug group × day of testing) as described previously when using a similarly designed study [33]. The three-way ANOVA revealed significant memory deficits due to LPS (*F*(1, 296) = 47.057, *P* < 0.001) and improvements related to drug treatment (*F*(2, 296) = 5.050, *P* = 0.007) and day (*F*(3, 296) = 45.838, *P* < 0.001). A *post hoc* two-way RM-ANOVA revealed significant main effects due to the infusion + drug group (*F*(5, 222) = 5.150, *P* < 0.001) and improvements related to day (*F*(3, 222) = 86.911, *P* < 0.001). LPS + vehicle rats were significantly impaired compared to aCSF + vehicle rats on days 2 to 4 (*P* < 0.05). Nimodipine treatment reversed LPS-induced deficits on days 3 and 4; specifically, LPS + nimodipine-treated rats took significantly (*P* < 0.05) shorter paths than LPS + vehicle rats and took paths that were not significantly (*P* > 0.150) different than their aCSF controls on days 3 and 4. Dantrolene treatment did not improve LPS-induced deficits, as LPS + dantrolene rats took significantly longer paths than their controls on all the days of testing (*P* < 0.05) and were not significantly different from the LPS + vehicle rats (*P* > 0.05). We found that LPS-treated rats had significantly faster swim speeds (Figure 1B; *F*(1, 74) = 29.173, *P* < 0.001) and *post hoc* analysis showed that every LPS group swam faster than their respective aCSF control (*P* < 0.01). Despite their increased swimming speed, LPS-infused rats also took more time to locate the hidden platform (*F*(1, 296) = 33.340, *P* < 0.001), as measured by latency to locate the hidden platform. Significant improvements in latency were observed related to drug treatment (*F*(2, 296) = 5.043, *P* = 0.007) and day of testing (*F*(3,296) = 80.392, *P* < 0.001). Similar to the results obtained with distance taken to locate the hidden platform (Figure 1C). A *post hoc* two-way RM-ANOVA revealed that the LPS + vehicle rats took significantly (*P* < 0.05) more time than the aCSF + vehicle rats to locate the hidden platform on days 2 to 4. Nimodipine treatment completely reversed LPS-induced deficits; specifically, the LPS + nimodipine-treated rats took significantly (*P* < 0.05) less time than the LPS + vehicle rats and were not significantly different (*P* > 0.05) from their aCSF controls on days 2 to 4. Dantrolene treatment led to a partial recovery of the LPS-induced deficit; specifically, the LPS + dantrolene-treated rats were not significantly different (*P* > 0.07) from their aCSF controls on day 4. There was no difference between any of the groups on day 1 (*P* > 0.05), suggesting that baseline performance was not driving the performance differences observed on the subsequent days of testing. There was also no difference between the aCSF groups on any day (*P* > 0.05), indicating that neither dantrolene nor nimodipine have cognitive enhancing effects or side effects that are detrimental to learning. For the probe trial, a two-way ANOVA revealed a significant main effect of infusion group (*F*(1, 76) = 4.093, *P* = 0.047). Overall, poor performance in the MWM was correlated with expression of the CaV1.2 subunit of the L-VDCC (*P* < 0.05 by Pearson correlation). Overall, these data indicate that LPS-induced spatial memory deficits in the MWM can be reversed by blockade of L-VDCCs, while blockade of RyRs only confers a minor improvement.

#### Blockade of L-VDCCs or RyRs during chronic neuroinflammation reduces aberrant expression of *Arc*

**Figure 2:**
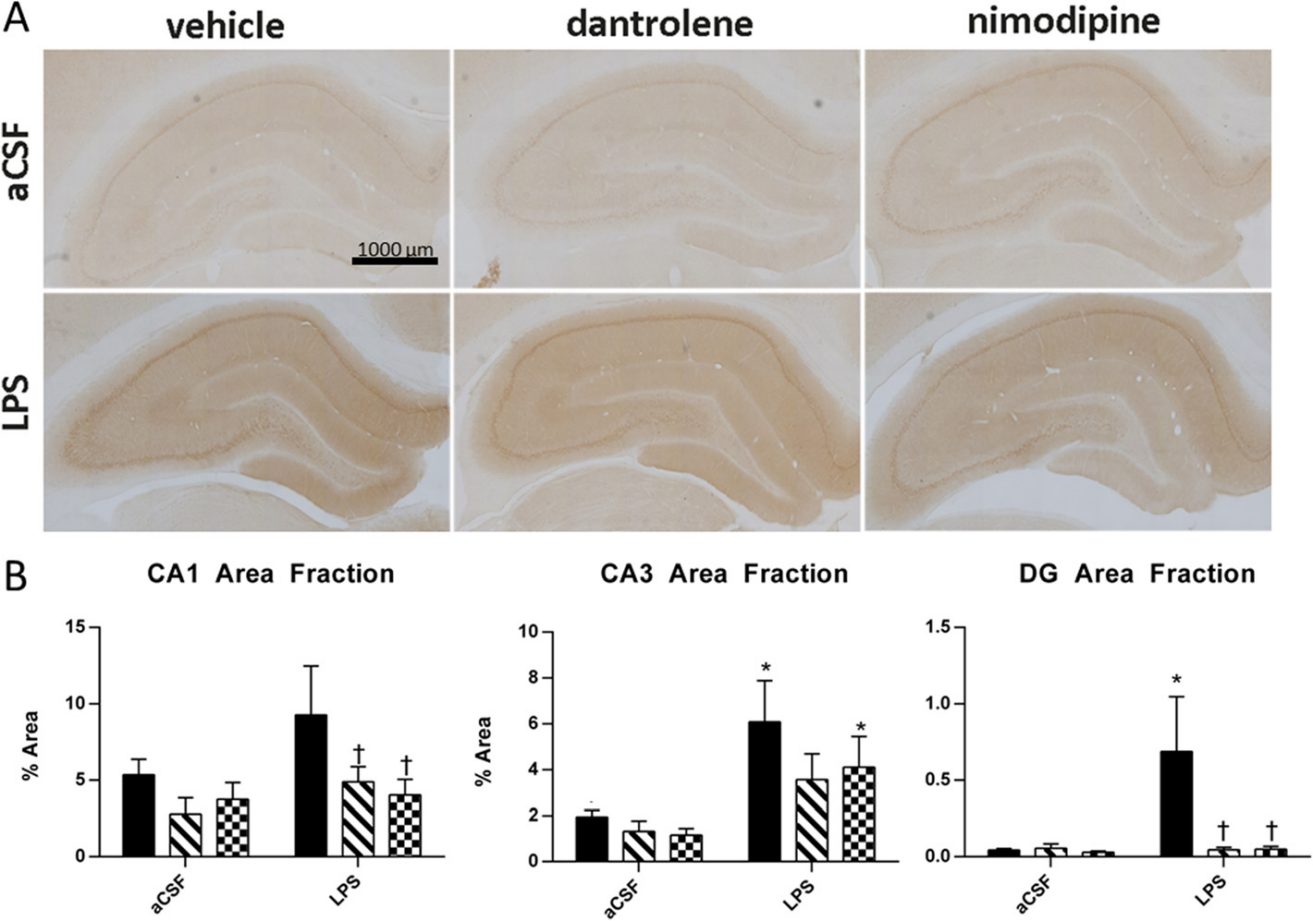
**Hippocampal immunohistochemistry against Arc was quantified across in brains perfused 30 min after rats were exposed to a novel context. (A)** Representative slices of hippocampal Arc immunohistochemistry after 4 weeks of infusion with aCSF (top row) or LPS (bottom row) and treatment with vehicle (first column), dantrolene (middle column), or nimodipine (third column). **(B)** Quantification of Arc immunostaining in CA1, CA1, and DG. Data expressed as mean ± SEM. *Indicates a significant difference from treatment-matched aCSF controls, ^†^indicates a significant difference from LPS + vehicle rats within the LPS group. Significance determined by *P* < 0.05. *LPS* lipopolysaccharide, *aCSF* artificial cerebrospinal fluid, *DG* dentate gyrus.

Due to the spatial memory improvements we observed in LPS-infused rats treated with drugs that normalize or reduce intracellular Ca^+2^ concentration, we examined levels of the IEG *Arc*, which is Ca^+2^ dependent. We examined expression of *Arc* protein in brains perfused 30 min after exposure to a novel spatial environment and found region-specific changes in levels as measured by immunohistochemistry and densitometry (Figure 2A, B). A two-way ANOVA revealed that *Arc* expression in the CA3 subfield of the hippocampus was increased by LPS infusion (*F*(1, 129) = 14.614, *P* < 0.001) and trended toward a main effect in the dentate gyrus (DG) subfield (*F*(1, 129) = 3.712, *P* = 0.056), with a significant LPS and drug interaction in the DG (*F*(2, 129) = 3.576, *P* = 0.031). In the DG, treatment with dantrolene or nimodipine reduced LPS-induced overexpression of *Arc* (*P* = 0.001 and *P* = 0.002, respectively) to aCSF levels. In the CA3, dantrolene treatment led to a slight reduction in LPS-induced *Arc* overexpression, and nimodipine treatment significantly reduced *Arc* overexpression. In the CA1, significant changes in *Arc* were related to drug treatment (*F*(1, 129) = 5.043, *P* = 0.007). The CA1 of vehicle-treated LPS-infused rats showed a trending increase in *Arc* (*P* = 0.079) compared to aCSF controls, which was significantly reduced by treatment with dantrolene or nimodipine (*P* = 0.050 and *P* = 0.027, respectively). Overall, chronic neuroinflammation increased expression of *Arc* and reduction of intracellular Ca^+2^ via L-VDCC or RyR blockade reduced these increases.

### Ca^+2^ dysregulation during chronic neuroinflammation is dependent on L-VDCC activity

**Figure 3:**
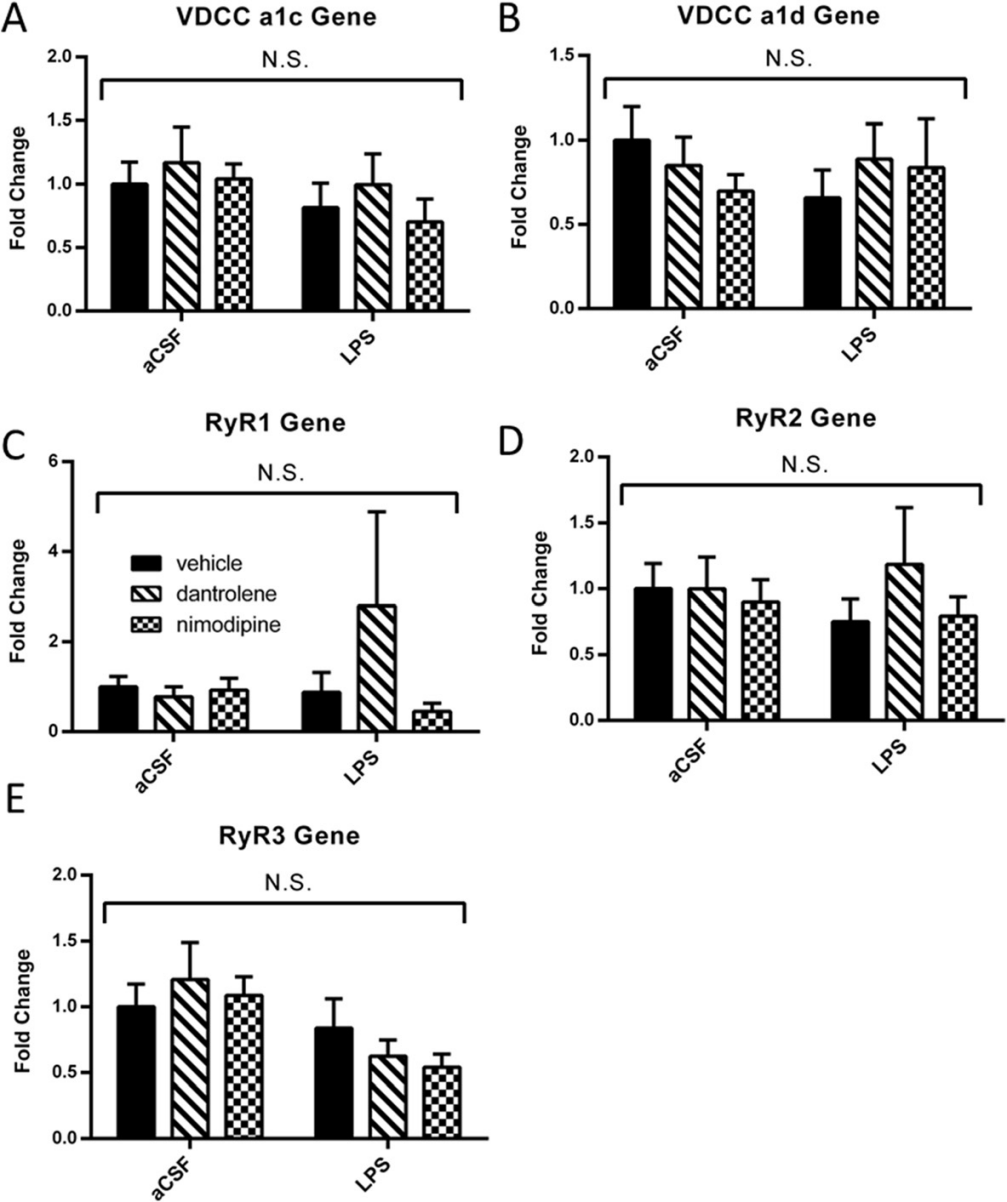
**Chronic LPS infusion did not change gene expression of either L-VDCC subunits or any of the RyR isoforms (A, B, C, D, E).** Data expressed as mean ± SEM. N.S. indicates no significant differences. Significance determined by *P* < 0.05. *LPS* lipopolysaccharide, *aCSF* artificial cerebrospinal fluid, *VDCC* voltage-dependent Ca^+2^ channel, *RyR* ryanodine receptor.

We hypothesized that chronic LPS infusion was triggering Ca^+2^ dysregulation, since we found that LPS infusion increases the Ca^+2^-dependent immediate early gene *Arc* and that increase is blocked by Ca^+2^ channel blockers. Ca^+2^ dysregulation in aging and AD has been associated with increases in L-VDCCs or RyRs [10,34-36]. Here, we examined hippocampal gene expression levels of the L-VDCC subunits a1c (CaV1.2) and a1d (CaV1.3), both of which are pharmacologically blocked by nimodipine, and found no significant differences (*P* > 0.05) across any LPS or drug treatments (Figure 3A, B). We also examined gene expression of the three RyR isoforms and found no significant differences (*P* > 0.05) across any LPS or drug treatments (Figure 3C, D, E).

**Figure 4:**
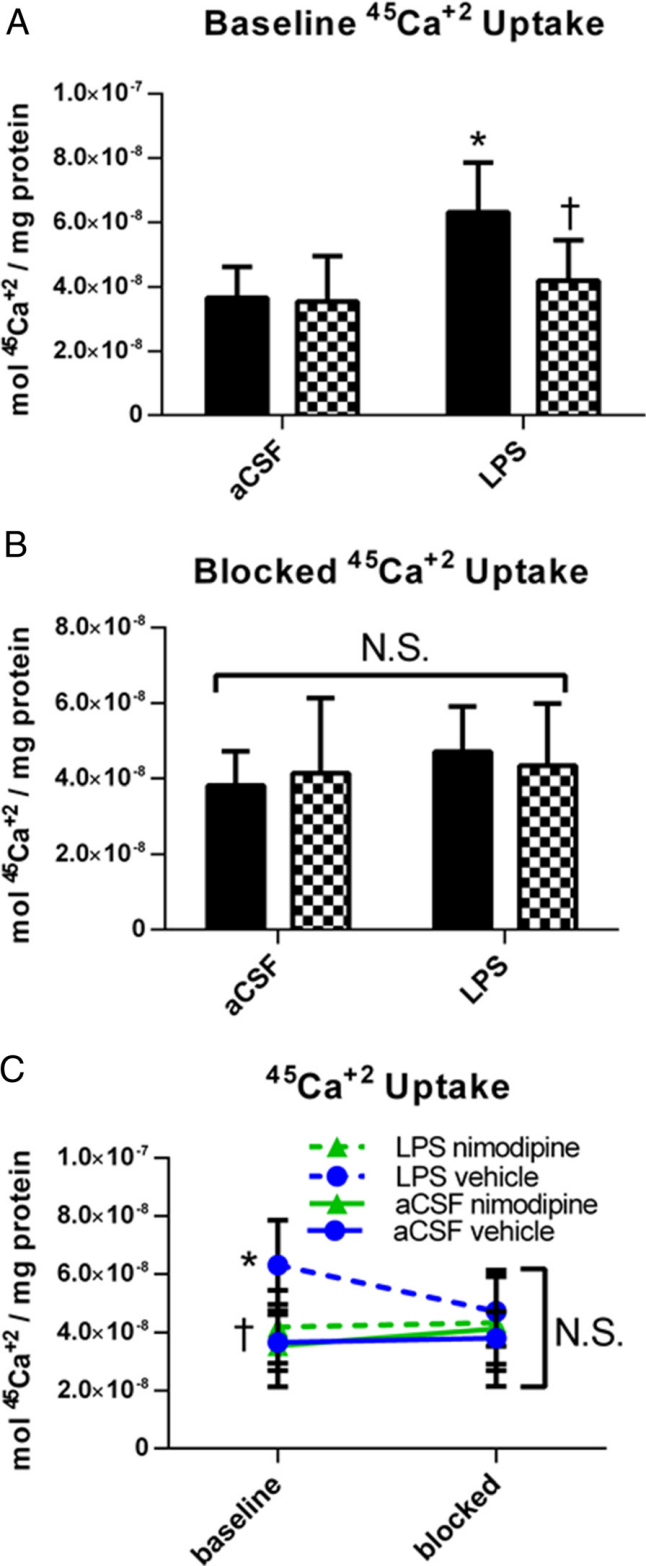
**Chronic LPS infusion increased hippocampal synaptosomal uptake of** ^**45**^ **Ca** ^**+2**^ **and this increase was reduced by treatment with nimodipine** ***in vivo*** **(A) or** ***ex vivo*** **(B and C).** (A) Baseline ^45^Ca^+2^ uptake by hippocampal synaptosomes isolated from rats infused with LPS or aCSF for 4 weeks and treated with vehicle or nimodipine. (B) ^45^Ca^+2^ uptake by hippocampal synaptosomes isolated from rats infused with LPS or aCSF for 4 weeks and treated with vehicle or nimodipine and additionally treated with *ex vivo* application of 30 μM nimodipine. (C) Data from figures (A) and (B) for comparison of baseline uptake and blocked uptake of ^45^Ca^+2^ with *ex vivo* nimodipine. Data expressed as mean ± SEM. *Indicates a significant difference from treatment-matched aCSF controls, ^†^Indicates a significant difference from LPS + vehicle rats, N.S. indicates no significant differences. Significance determined by *P* < 0.05. *LPS* lipopolysaccharide, *aCSF* artificial cerebrospinal fluid.

We tested whether chronic neuroinflammation would functionally increase intracellular Ca^+2^ uptake by generating synaptosomes from fresh hippocampus from rats infused with chronic LPS or aCSF and treated with vehicle or nimodipine and measuring ^45^Ca^+2^ uptake using liquid scintillation (Figure 4). At baseline (Figure 4A), a two-way ANOVA revealed a significant main effect of drug (*F*(1, 34) = 4.410, *P* = 0.043) and a trend toward a group main effect (*F*(1, 34) = 3.169, *P* = 0.084). *Post hoc* analyses revealed that the LPS + vehicle rats had significantly more moles of ^45^Ca^+2^ uptake per mg of protein compared to the aCSF + vehicle rats (*P* = 0.034) as well as compared to the LPS + nimodipine rats (*P* = 0.026). The LPS + nimodipine rats were no different from the aCSF + nimodipine rats or aCSF + vehicle rats (*P* > 0.5). These results demonstrated that increases in Ca^+2^ were dependent on L-VDCC activity, since nimodipine was able to reverse LPS-associated increases in Ca^+2^ uptake. In order to elucidate the role of L-VDCCs, we applied 10 μM of nimodipine *ex vivo* to the same hippocampal synaptosomal preparations (Figure 4B). With *ex vivo* nimodipine, there were no differences across any LPS or drug treatments (*P* > 0.2). When we examined the data with a three-way ANOVA across the two conditions (baseline and blocked, Figure 4C), we found a significant main effect of group (*F*(1, 67) = 4.73, *P* = 0.033) and drug (*F*(1, 67) = 5.794, *P* = 0.019), but not between conditions (*F*(1, 67) = 0.655, *P* = 0.421). Overall, these data demonstrate that there is Ca^+2^ dysregulation during neuroinflammation that is dependent on activity of L-VDCCs.

### L-VDCC or RyR antagonism reduces hippocampal neuroinflammatory markers during chronic neuroinflammation

**Figure 5:**
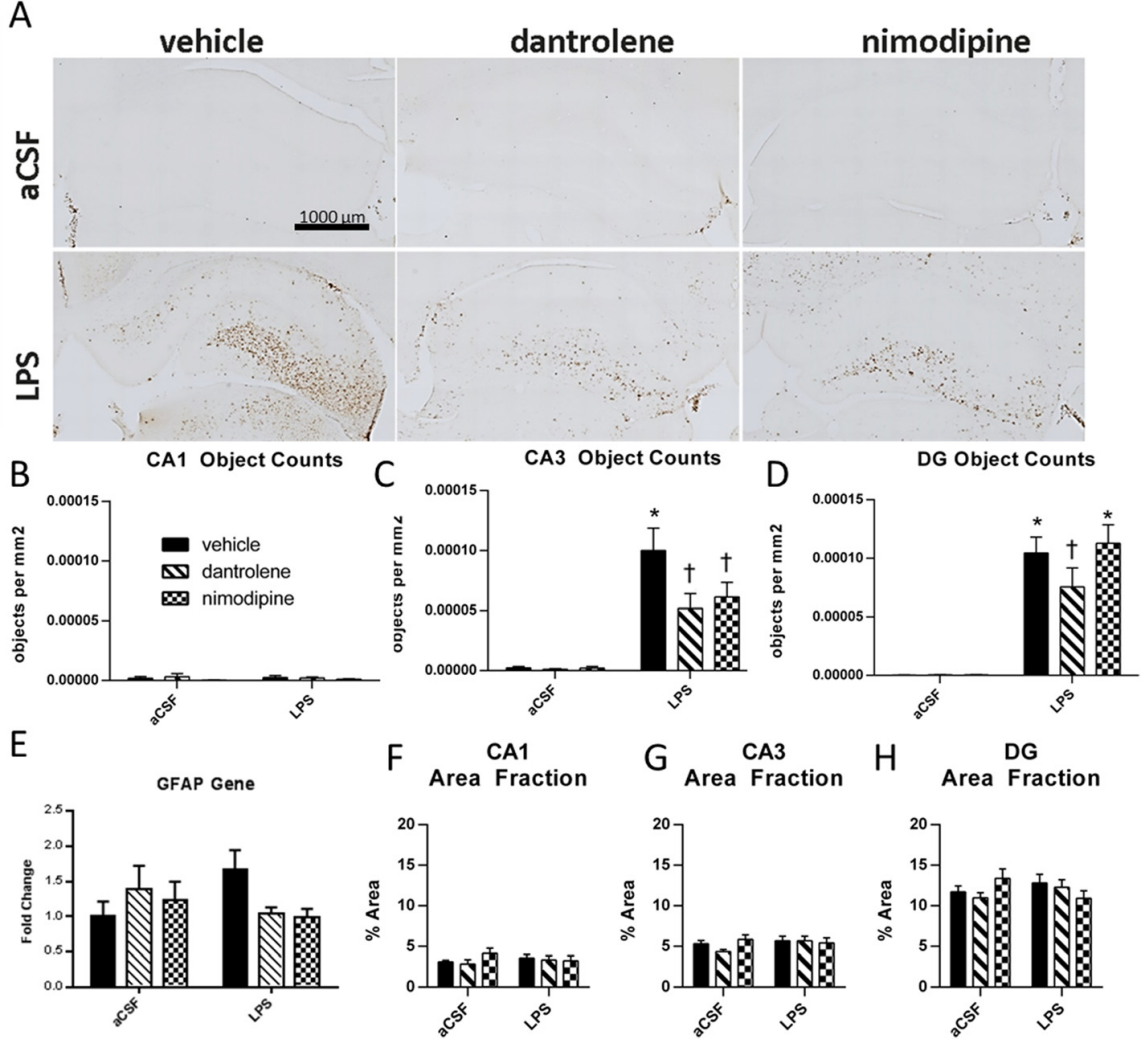
**Chronic LPS infusion increased activation of microglia but not astrocytes, and treatment with either dantrolene or nimodipine reduced microglia activation.** Activated microglia were quantified by counting MHC-II+ cells, and activated astrocytes were quantified by densitometry of GFAP. Hippocampal immunohistochemistry against MHC-II/OX-6 **(A)** and GFAP was quantified **(B, C, D, F, G, H)** across specific hippocampal subfields. Although the DG region in LPS-infused rats contains numerous immunoreactive objects, only those OX-6 immunoreactive objects larger than 65 mm^2^ were included in analysis and are represented in the histograms. GFAP hippocampal gene expression **(E)** was also quantified. (B) There was no change in the number of MHC-II+ cells in the CA1 subfield of the hippocampus. (C, D) There were significantly more MHC-II positive cells in the CA3 (C) and DG (D) subfield of all LPS-treated groups compared to their aCSF controls. Dantrolene treatment significantly reduced the number of MHC-II+ cells present in the CA3 and DG subfields, and nimodipine treatment significantly reduced the number of MHC-II+ cells present in the CA3. (E) There was a trend toward a drug × group interaction for hippocampal GFAP gene expression, but no statistically significant changes were observed. (F, G, H) There were no significant changes in the amount of GFAP in any of the hippocampal subfields due to either LPS or drug treatment. Data expressed as mean ± SEM. *Indicates a significant difference from treatment-matched aCSF controls, ^†^Indicates a significant difference from LPS + vehicle rats. Significance determined by *P* < 0.05. *LPS* lipopolysaccharide, *aCSF* artificial cerebrospinal fluid, *DG* dentate gyrus, *GFAP* glial fibrillary acid protein.

We evaluated the role of L-VDCC and RyR antagonism on LPS-induced microglia activation in the hippocampus by performing immunohistochemistry against MHC-II (Figure 5A). Numerous activated microglia were present throughout the hippocampi of LPS-infused rats, with few present in aCSF-infused rats. MHC-II positive (MHC-II+) cells were counted in specific hippocampal subfields (Figure 5B, C, D). Very few activated microglia were present in the CA1 subfield (Figure 5A), with no differences across any of the groups (*P* > 0.3). The CA3 and DG (Figure 5C, D) subfields had more MHC-II+ cells, and two-way ANOVAs revealed a significant main effect of drug and group in the CA3 (*F*(2, 122) = 3.069, *P* = 0.050 and *F*(1, 122) = 33.642, *P* < 0.001, respectively) and a significant main effect of group in the DG (*F*(2, 122) = 91.203, *P* < 0.001). *Post hoc* analyses on the CA3 and DG showed that LPS-infused rats treated with vehicle, dantrolene, or nimodipine all had significantly more MHC-II positive cells compared to their aCSF controls (*P* < 0.02). In the CA3, dantrolene- and nimodipine-treated LPS rats had significantly fewer MHC-II+ cells compared to vehicle-treated LPS rats (*P* < 0.001 and *P* = 0.011, respectively). In the DG, the LPS + dantrolene rats had fewer MHC-II+ cells than the LPS + vehicle rats (*P* = 0.028), but the LPS + nimodipine rats were not significantly different (*P* > 0.1). Overall, the blockade of RyRs and L-VDCCs reduces hippocampal microglia activation induced by chronic LPS infusion. Furthermore, changes in multiple biomarkers in response to drug treatment were likely more robust in the dentate and CA3 regions of the hippocampus, as compared to the CA1 region, due to the fact that these regions typically demonstrate a more robust inflammatory response to chronic LPS infusion [6].

We then assessed astrocyte activation after chronic LPS infusion by examining hippocampal GFAP gene expression (Figure 5E) and quantified GFAP immunohistochemistry in hippocampal subfields (Figure 5F, G, H). A two-way ANOVA revealed a trend toward interaction between drug and group (*P* = 0.057) on hippocampal GFAP gene expression, but there were no significant differences between any of the groups. Similarly, immunohistochemistry quantification revealed no significant differences in the CA1 (Figure 5F), CA3 (Figure 5G), or DG (Figure 5H), although there was a trend toward significant interaction of drug and group on DG GFAP expression (*P* = 0.082). Taken together with the MHC-II immunohistochemistry data, these data demonstrate a more significant role for microglia than astrocytes in LPS-induced neuroinflammation.

**Figure 6:**
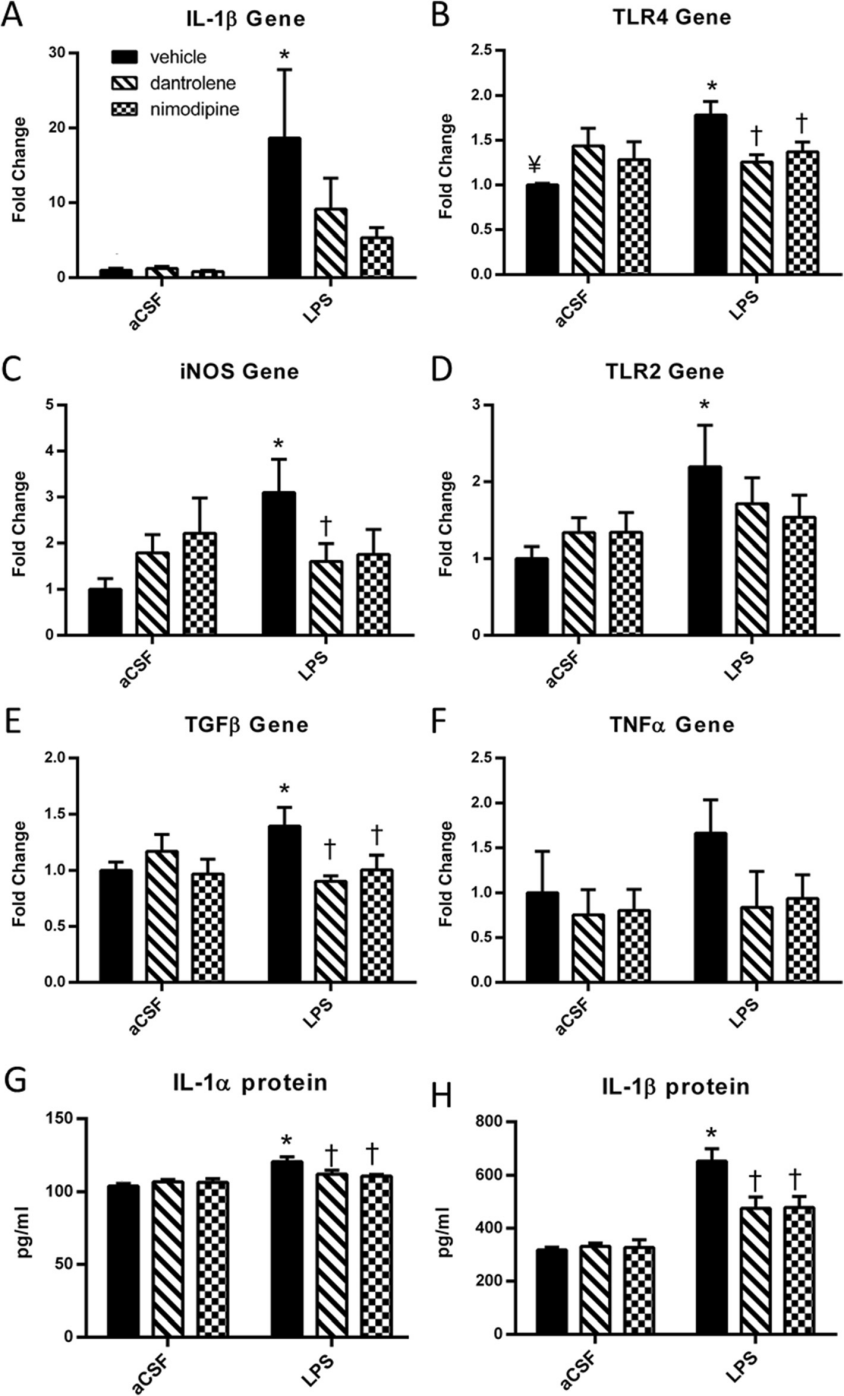
**Chronic LPS infusion increased gene and protein expression of several inflammatory markers and these increases were reduced by treatment with either dantrolene or nimodipine.** Gene expression of IL-1β **(A)**, TLR4 **(B)**, iNOS **(C)**, TLR2 **(D)**, and TGFβ **(E)** was significantly increased in LPS + vehicle rats compared to aCSF + vehicle rats. In LPS rats, treatment with nimodipine significantly reduced expression of TLR4 mRNA (B), TGFβ mRNA (E), IL-1α protein **(G)**, and IL-1β protein **(H)** and led to a trend in reduction of the IL-1β gene ((A), *P* = 0.053) and iNOS gene ((C), *P* = 0.085). In LPS rats, treatment with dantrolene significantly reduced the expression of TLR4 gene (B), iNOS gene (C), TGFβ gene (E), IL-1α protein (G), and IL-1β protein (H). There was no significant change in TNFα gene expression **(F)**. Data expressed as mean ± SEM. *Indicates a significant difference from treatment-matched aCSF controls, ^†^Indicates a significant difference from LPS + vehicle rats, ^¥^Indicates significant difference from aCSF + dantrolene rats. Significance determined by *P* < 0.05. *LPS* lipopolysaccharide, *aCSF* artificial cerebrospinal fluid, *TLR4* Toll-like receptor 4, *TLR2* Toll-like receptor 2, *iNOS* inducible nitric oxide synthase, *TGFβ* transforming growth factor β, *TNFα* tumor necrosis factor alpha.

We further elucidated the neuroinflammatory milieu of the hippocampus following chronic LPS infusion by evaluating several inflammatory genes in whole hippocampal homogenate (Figure 6). Two-way ANOVA revealed that IL-1β and Toll-like receptor 2 and 4 (TLR2 and TLR4) gene expression was significantly affected by the LPS vs. aCSF group (*F*(1, 40) > 4, *P* < 0.05), and TLR4, inducible nitric oxide synthase (iNOS), and transforming growth factor β (TGFβ) gene expression was significantly affected by an interaction between drug and group (*F*(2, 40) > 3.5, *P* < 0.05). *Post hoc* analyses revealed that the LPS + vehicle rats had significant (*P* < 0.05) increases in gene expression of IL-1β, TLR4, iNOS, TLR2, and TGFβ compared to the aCSF + vehicle rats. Treatment with nimodipine decreased the gene expression of IL-1β, TLR4, iNOS, and TGFβ (*P* = 0.053 trend, *P* = 0.039, *P* = 0.085 trend, and *P* = 0.033, respectively), while treatment with dantrolene decreased gene expression of TLR4, iNOS, and TGFβ (*P* = 0.007, *P* = 0.050, and *P* = 0.006, respectively). For IL-1β, iNOS, and TLR2 expression, dantrolene or nimodipine-treated LPS rats that did not reach *P* < 0.05 were not significantly different than their aCSF controls or LPS + vehicle rats, indicating an intermediate reduction with drug treatment.

## Discussion

### Nimodipine and dantrolene differentially improved memory deficits associated with LPS infusion

Nimodipine treatment resulted in a complete recovery of spatial memory deficits induced by chronic LPS infusion as measured by performance in the MWM. These data implicate an important role of Ca^+2^ overloads in neuroinflammation-induced memory deficits. The mechanism by which nimodipine improves spatial memory may be by reduction of the slow after hyperpolarization (sAHP). Reduction of the sAHP is correlated with improved memory acquisition [37]. This would improve neuronal sensitivity to relevant event-related stimuli, which may be overshadowed by Ca^+2^ “noise” during neuroinflammation. Indeed, blockade of tumor necrosis factor alpha (TNFα) signaling during aging reduces age-related increases in the sAHP and improves memory [38], suggesting a relationship between neuroinflammation, memory, and the enhancement of L-VDCCs. Similarly, increased RyR dysregulation also underlies age-associated Ca^+2^ dysregulation and increases in the sAHP. In young rats, *in vitro* RyR blockade reduces the sAHP to a lesser extent compared to L-VDCC antagonism [39]. Aged neurons demonstrate prolonged increases in intracellular Ca^+2^ levels that are RyR-dependent [40]. Oxidative stress present in aged rats increases the sAHP by 50% and is dependent on RyRs, but not other sources of Ca^+2^, including inositol triphosphate receptors and L-VDCCs [36]. Additionally, L-VDCCs and RyRs interact with each other: Ca^+2^ influx via L-VDCCs triggers Ca^+2^-induced Ca^+2^ release via RyRs which in turn modulates L-VDCC activity ([41];). Furthermore, a recent study on patients with late onset AD demonstrated a genetic interaction between L-VDCC and RyR mutations and amyloid deposition [11], demonstrating the importance of these two channels in the AD pathology.

In the present study, dantrolene treatment did not improve memory to the same extent as nimodipine. However, dantrolene was still able to normalize many biochemical changes induced by chronic LPS infusion to the same extent as nimodipine. This discrepancy may be accounted for by dantrolene’ s interaction with NMDAR function: RyR activation is important for the amplification of NMDAR signals [26,27]. While reduction of the sAHP by nimodipine and dantrolene may increase relevant NMDAR signaling, dantrolene may also reduce relevant NMDAR signaling, leading to a disruption of memory performance. On the surface, this concept may be in conflict with our previous observation that NMDAR blockade with the noncompetitive antagonist memantine improves memory during chronic neuroinflammation [32], but memantine’ s kinetics allow for relevant signals to pass through [42], making its mechanism of neuroprotective and nootropic effects more similar to nimodipine.

### Dantrolene and nimodipine normalize LPS-induced increases in Arc and intracellular Ca^+2^

During chronic LPS infusion, *Arc* expression is increased in the hippocampus, which parallels our previous studies [32]. While *Arc* is required for late-LTP and memory consolidation and *Arc* deficiency leads to memory deficits [43], under normal circumstances, its expression is sparse and specific [44], similar to electrophysiological changes that take place during learning and memory. Overexpression of *Arc* is most likely not beneficial to memory during chronic neuroinflammation. *Arc* expression leads to endocytosis of AMPA receptors (AMPAR) [45] and a decrease in AMPAR-mediated excitation and induction of long-term depression (LTD; [46,47]). Normally, the function of this may be to maintain homeostatic synaptic scaling [48] and would be neuroprotective by decreasing glutamatergic postsynaptic activity. However, an extended increase in *Arc* could cause a protracted decrease in synaptic excitability or LTD that could eventually lead to increased synaptic elimination [49].

Treatment with dantrolene or nimodipine reduced LPS-induced *Arc* increases. The mechanism by which dantrolene and nimodipine reduce *Arc* expression may be due to a reduction in LPS-induced increases of intracellular Ca^+2^. Indeed, *Arc* expression is known to be Ca^+2^-dependent [29]. Several lines of evidence suggest that there is Ca^+2^ dysregulation during neuroinflammation. For one, neuroinflammation has been shown to reduce glutamate uptake by [16,18] and potentiate glutamate release from [50,51] glial cells. NMDAR blockade by memantine has been shown to reduce *Arc* expression during chronic neuroinflammation [32], further supporting this concept. Potentiated Ca^+2^ entry via L-VDCCs and RyRs by cytokines and other pro-inflammatory markers have also been observed ([22,23,33]. By restoring normal intracellular Ca^+2^ levels, dantrolene and nimodipine may prevent LPS-induced overexpression of *Arc*. Indeed, LPS-infused rats had significantly increased ^45^Ca^+2^ uptake. Previously, Ca^+2^ dysregulation induced by the pro-inflammatory cytokine IL-1β has been observed directly using Ca^+2^ imaging *in vitro* [52]. To the best of our knowledge, this is the first study to directly document Ca^+2^ dysregulation following *in vivo* chronic neuroinflammation as opposed to *in vitro* acute neuroinflammation. Strikingly, nimodipine treatment *in vivo* or *ex vivo* was able to reverse completely LPS-induced increases in ^45^Ca^+2^ uptake. These data demonstrate that the L-VDCC blockade is sufficient to reverse LPS-induced Ca^+2^ dysregulation.

### Potential mechanisms by which dantrolene and nimodipine reduce neuroinflammation

Dantrolene and nimodipine dramatically reduce the number of activated microglia in the hippocampus and reduce the expression of various pro-inflammatory cytokines. It is not clear from our data whether the anti-inflammatory effects of dantrolene and nimodipine are due to direct action on the microglia themselves or an indirect effect via normalization of neuronal Ca^+2^ levels. Neurotoxicity of conditioned media from activated microglia is reduced when drugs blocking L-VDCCs or RyRs are applied to the microglia cultures [12-14]. However, the *in vivo* anti-inflammatory effects of these drugs are not so clear-cut. Following facial nerve transection, nimodipine treatment improves motor neuron survival without reducing microglia activation [53]. However, after ischemic-reperfusion injury, nimodipine does improve behavioral outcomes while concurrently reducing microglia activation [54]. Similarly, *in vivo* treatment with dantrolene is neuroprotective and improves behavioral outcomes in various *in vivo* models of chronic neurodegenerative disorders such as Huntington’s [55], AD [56,57], and spinocerebellar ataxia [58]. Chronic neuroinflammation is a pathological component in all of these disorders [59-61]. However, these studies did not examine microglia activation, making it difficult to determine whether modulation of neuroinflammation played a role in the improvement garnered by nimodipine and dantrolene in these studies.

These drugs may act by directly reducing microglia activation. Intracellular Ca^+2^ is directly involved in microglia activation. Ca^+2^ is required for LPS-mediated microglia activation *in vitro*, with application of a Ca^+2^ chelator sufficient to prevent activation and production of pro-inflammatory species [62]. RyRs and L-VDCCs may be involved in mediating Ca^+2^-associated microglia activation. Microglia express mRNA of the RyR1 and RyR2 subtypes and application of a RyR antagonist prevents LPS-induced neurotoxicity mediated by microglia [14], suggesting a direct role of RyRs in microglia activation. On the other hand, L-VDCC expression on microglia is still debated; the *in vitro* anti-inflammatory effect of nimodipine may be mediated by off-target effects of nimodipine [12]. Specifically, nimodipine may act by inhibiting the microglia NOX pathway directly, resulting in reduced superoxide production [13]. Earlier studies showed that activation of L-VDCCs increased superoxide production as well as Ca^+2^ influx in microglia, which could be blocked by nifedipine, a drug closely related to nimodipine [63]. Regardless of whether microglia express functional L-VDCCs, it is possible that nimodipine is exerting direct anti-inflammatory effects during chronic LPS infusion.

L-VDCC blockers, such as dantrolene and nimodipine, can also relax vascular smooth muscle by inhibiting Ca^+2^ influx leading to vasodilation in the presence of cerebral vasospasms induced by subarachnoid hemorrhage that can lead to brain ischemia, oxidative stress, and neuroinflammation [64]. However, the vascular actions of these drugs most likely do not underlie their beneficial effects in the current study since LPS exposure upregulates the inducible form of nitric oxide synthase leading to an elevated release of nitric oxide [65] and subsequent vasodilation.

Microglia monitor the status of nearby neurons [66] via a variety of channels and receptors. Importantly, microglia can sense depolarization of nearby neurons via K+ channels [67]. The data herein show that dantrolene and nimodipine are both capable of reducing LPS-induced increases in hippocampal *Arc* expression. Because *Arc* expression requires intracellular Ca^+2^ [29], it makes sense that these drugs are directly reducing intraneuronal Ca^+2^ levels, ostensibly reducing neuronal depolarization, which may in turn reduce activation of nearby microglia. Furthermore, neurons suffering from Ca^+2^ overload are known to alert microglia by release of chemokines [68]. Therefore, reduction of neuronal Ca^+2^ by nimodipine and dantrolene may prevent activation of microglia by neuronal-mediated mechanisms. Here, we did not observe any effect of LPS or drug treatment on expression of CD200 receptor or ligand (data not shown), indicating that upregulation of CD200 ligand on neurons is not mediating an anti-inflammatory feedback mechanism similar to that which is suggested above. Regardless of the specific mechanism, these data suggest that treatment with dantrolene or nimodipine is sufficient to break the self-propagating cycle of neuroinflammation.

## Conclusions

Neuroinflammation drives a self-propagating feed forward cycle, where activated microglia release cytokines and NO that are injurious to neurons, and injured neurons release factors that also activate nearby microglia. Cytokines also feed back onto microglial cytokine receptors, triggering activation of additional nearby microglia. Our model of chronic LPS infusion triggers this cascade: after cessation of LPS infusion, neuroinflammation and memory deficits persist after 5 weeks [29], suggesting that even without LPS present, microglia that have already been activated continue to maintain a pro-inflammatory environment. The ability of dantrolene and nimodipine to disrupt this cycle suggests that Ca^+2^ dysregulation is a viable target for interrupting the cycle of neuroinflammation that may contribute to neurodegenerative diseases such as AD.
